# Draft Genome Sequence of *Bacillus thuringiensis* Strain BrMgv02-JM63, a Chitinolytic Bacterium Isolated from Oil-Contaminated Mangrove Soil in Brazil

**DOI:** 10.1128/genomeA.01264-13

**Published:** 2014-01-30

**Authors:** Joelma Marcon, Rodrigo Gouvêa Taketani, Francisco Dini-Andreote, Giulia Inocêncio Mazzero, Fabio Lino Soares, Itamar Soares Melo, João Lúcio Azevedo, Fernando Dini Andreote

**Affiliations:** a Department of Genetics, Luiz de Queiroz College of Agriculture, University of São Paulo, Piracicaba, São Paulo, Brazil; b Laboratory of Environmental Microbiology, EMBRAPA Environment, Jaguariúna, São Paulo, Brazil; c Department of Microbial Ecology, Centre for Ecological and Evolutionary Studies, University of Groningen, Groningen, The Netherlands; d Department of Soil Sciences, Luiz de Queiroz College of Agriculture, University of São Paulo, Piracicaba, São Paulo, Brazil

## Abstract

Here, we report the draft genome sequence and the automatic annotation of *Bacillus thuringiensis* strain BrMgv02-JM63. This genome comprises a set of genes involved in the metabolism of chitin and *N*-acetylglucosamine utilization, thus suggesting the possible role of this strain in the cycling of organic matter in mangrove soils.

## GENOME ANNOUNCEMENT

Chitinases are enzymes that catalyze the conversion of chitin (a linear homopolysaccharide of β-1,4-*N*-acetylglucosamine) to its monomeric compounds. These enzymes are widely distributed in nature, being produced by a large variety of chitin-degrading organisms, including bacteria, fungi, insects, plants, and animals (1). The Gram-positive aerobic/facultative anaerobic endospore-forming bacterium *Bacillus thuringiensis* is commonly found in soils and estuarine sediments, as well as in association with plant roots (2, 3). *B. thuringiensis* strain BrMgv02-JM63 was originally isolated from oil-contaminated mangrove soil located in the city of Bertioga, São Paulo, Brazil (23°53′49″S, 46°12′28″W). This bacterium presents the ability to solubilize chitin when growing in minimum medium amended with 1% colloidal chitin. Features related to chitinolytic activity have been annotated in the draft genome.

Shotgun sequencing of the *B. thuringiensis* BrMgv02-JM63 genome was performed using the Ion 316 chip technology provided in the Ion sequencing kit 200 version 2.0, according to the manufacturer’s protocol. The genome sequence was *de novo* assembled using MIRA version 3.4, CLC Genomics Workbench version 5.5.1, and SOAP*denovo*2 assembler (4). The obtained contigs were further integrated using CISA (5).

A total of 3,106,906 reads (*Q* > 20), with a mean length of 145 bp, were assembled using a reference-based approach and allocated into 33 contigs ranging from 32,627 to 545,251 bp in length. The mean G+C content of the genome is 35%, and genome coverage depth is approximately 90×. The assembled data were automatically annotated by RAST (6). The draft genome size is 4,931,802 bp, comprising 5,137 open reading frames (ORFs) and 51 RNA genes. Automatic annotation by RAST predicted a total of 18 genes involved in the metabolism of chitin and *N*-acetylglucosamine (NAG) utilization. These predicted genes account for one copy of the regulator *nagR*, two copies of the gene *nagA*, one copy each of the *nagB1*, *nagEa*, *nagEb*, *nagEc*, and *nagQ* genes, seven copies of chitinase (ChiA_20) (EC 3.2.1.14), and three copies of chitin binding protein.

*Bacillus* spp. have been described as chitin degraders in soils (7, 8) and phylloplane (9) and in association with insects (10). However, the particular conditions found in mangrove soils (i.e., low oxygen availability and salinity) might select features unique to chitinases that have evolved in this environment. The ongoing work will quantify the efficiency of this strain in degrading *in vitro* colloidal chitin, and also the genomic arrangement of these genes will be compared across closely related genomes publicly available in the database.

### Nucleotide sequence accession numbers.

The *B. thuringiensis* strain BrMgv02-JM63 genome sequence and annotation data have been deposited at DDBJ/EMBL/GenBank under the accession no. AYSM00000000. The version described in this paper is version AYSM01000000.
